# Integrating machine learning and single-cell trajectories to analyze T-cell exhaustion to predict prognosis and immunotherapy in colon cancer patients

**DOI:** 10.3389/fimmu.2023.1162843

**Published:** 2023-05-03

**Authors:** Xiaogang Shen, Xiaofei Zuo, Liang Liang, Lin Wang, Bin Luo

**Affiliations:** ^1^ Department of Gastrointestinal Surgery, Sichuan Provincial People’s Hospital, University of Electronic Science and Technology of China, Chengdu, China; ^2^ Chinese Academy of Sciences Sichuan Translational Medicine Research Hospital, Chengdu, China; ^3^ Cancer Center, Sichuan Provincial People’s Hospital, University of Electronic Science and Technology of China, Chengdu, China

**Keywords:** colon adenocarcinoma (COAD), t-cell exhaustion, tumor microenvironment, prognosis, immunotherapy

## Abstract

**Introduction:**

The incidence of colon adenocarcinoma (COAD) has recently increased, and patients with advanced COAD have a poor prognosis due to treatment resistance. Combining conventional treatment with targeted therapy and immunotherapy has shown unexpectedly positive results in improving the prognosis of patients with COAD. More study is needed to determine the prognosis for patients with COAD and establish the appropriate course of treatment.

**Methods:**

This study aimed to explore the trajectory of T-cell exhaustion in COAD to predict the overall survival and treatment outcome of COAD patients. Clinical data were derived from the TCGA-COAD cohort through "UCSC", as well as the whole genome data. Prognostic genes driving T-cell trajectory differentiation were identified on the basis of single-cell trajectories and univariate Cox regression. Subsequently, T-cell exhaustion score (TES) was created by iterative LASSO regression. The potential biological logic associated with TES was explored through functional analysis, immune microenvironment assessment, immunotherapy response prediction, and in vitro experiments.

**Results:**

Data showed that patients with significant TES had fewer favorable outcomes. Expression, proliferation, and invasion of COAD cells treated with TXK siRNA were also examined by cellular experiments. Both univariate and multivariate Cox regression indicated that TES was an independent prognostic factor in patients with COAD; in addition, subgroup analysis supported this finding. Functional assay revealed that immune response and cytotoxicity pathways are associated with TES, as the subgroup with low TES has an active immune microenvironment. Furthermore, patients with low TES responded better to chemotherapy and immunotherapy.

**Conclusion:**

In this study, we systematically explored the T-cell exhaustion trajectory in COAD and developed a TES model to assess prognosis and provide guidelines for the treatment decision. This discovery gave rise to a fresh concept for novel therapeutic procedures for the clinical treatment of COAD.

## Introduction

Colon adenocarcinoma (COAD) is a malignant gastrointestinal tumor that originates in the colon and is the third most prevalent tumor and the second leading cause of cancer-related death worldwide (1). Early-stage COAD is often difficult to detect, and despite rapid advances in early cancer screening technology, most COAD patients are diagnosed with advanced COAD only when they have obvious symptoms such as hematochezia and colonic obstruction, etc (2). Patients with advanced COAD are often not eligible for surgical resection and can only be treated with traditional chemotherapy and targeted therapy (Monoclonal antibody drugs such as bevacizumab and cetuximab) (3, 4). However, treatment resistance often occurs in advanced COAD, leading to a worse prognosis for patients (5). As cancer treatment has entered the field of immunotherapy, most cancer patients have achieved clinical success (6). However, only a small percentage of patients respond positively to immunotherapy.

Most patients with advanced cancer have T cells in a state of exhaustion, and T-cell exhaustion is an important factor in the efficacy of immunotherapy (7). T-cell exhaustion is a common feature of the cancer process and immune dysfunction, resulting from sustained antigenic stimulation and immune response (8). Exhausted T cells have a progressive loss of immune effector function, persistent high expression of suppressor receptors (such as ENTPD1, LAYN, LAG3, and HAVCR2) (9), and loss of self-renewal capacity, along with a unique transcriptional signature (8, 10). Recent studies suggest that interventions to alleviate T-cell exhaustion may lead to superior clinical outcomes and dramatic advances in cancer immunotherapy (11). Encouragingly, related studies have made some progress in lung cancer (12, 13). Thus, finding pre-depleted T cells in COAD could lead to a larger clinical window. Single-cell sequencing technology provides a new perspective for analyzing T-cell exhaustion trajectories (14), and by integrating scRNA-Seq data and bulk RNA-seq data, we may be able to gain a preliminary understanding of the T-cell trajectories and core regulatory targets that are pre-exhausted in COAD. This will facilitate initial protocol development and the development of novel targeted therapies.

Tumor processes are complex dynamic systems, and although it is commonly assumed that exhausted T cells result from sustained antigenic stimulation, the phenotype and transcriptional characteristics of exhausted T cells are also shaped by multiple factors in the immune microenvironment (15). Including the expression of suppressive receptors and ligands, the regulation of suppressor cells such as Tregs, suppressor cytokines such as IL-10 and TGFb, and some metabolic products also suppress T-cell function (15). Identification of the transcriptional pathways mediating T cell dysfunction is complex because the genetic profile of exhausted T cells overlaps to a considerable extent with that of activated T cells (16, 17). It has been suggested that failing T cells cannot be accurately defined by suppressor molecules alone (8), however, the driver genes that regulate T cell failure in COAD are currently unknown. These genes may be a breakthrough in targeting T-cell exhaustion and may also serve as important clinical prognostic treatment targets.

In this study, we aimed to characterize the dynamic trajectory of T-cell exhaustion in COAD and identify prognostic markers associated with exhausted CD8+ T cells. We first depicted the evolutionary trajectory of CD8+ T cells in a single-cell dataset and identified core regulatory genes of the exhausted CD8+ T trajectory based on the pseudo-time trajectory. Based on these genes we constructed a T-cell exhaustion score (TES) to assist in prognosis and quantify the degree of exhaustion. We then assessed the heterogeneity of different TES subgroups in terms of function, immune infiltration, and genomic alterations, and evaluated the predictive efficacy of the TES for immunotherapy. Finally, we preliminarily validated the core genes of TES by qRT-PCR, CCK8 assay, and transwell invasion experiment. In conclusion, this study not only identified the trajectory of exhausted CD8+ T cells in COAD and provided a tool to quantify T-cell exhaustion. Moreover, it confirmed the reliable efficacy of T-cell exhaustion in predicting COAD prognosis and immunotherapy. We hope that this study will provide novel prognostic markers and immunotherapeutic targets for COAD patients.

## Methods

### Data collection

For the TCGA-COAD cohort, we obtained copy number variant (CNV) data from the UCSC Xena (https://xena.ucsc.edu) database, somatic variant data from maf files on the Muctect 2 platform, and transcriptome RNA-seq data. Additionally, relevant clinical follow-up data was gathered. A TCGA-COAD cohort of 432 COAD patients was collected and utilized as a training cohort after patients with pathological typing as COAD were included and patients with missing follow-up information were excluded. Additionally, data from three large COAD cohorts: GSE14333 (18), GSE17536 (19), and GSE41258 (20) from the GEO database was gathered. After data combination and removing those with insufficient follow-up data, 654 patients with COAD were included after collecting the patient follow-up data from the original Supplementary Material. The external validation was done using the meta-GEO COAD cohort. Finally, a single-cell transcriptome dataset GSE146771 of 10 primary tumor sections, was obtained and processing use “Seurat” R packages. We explore T-cell exhaustion trajectories in colon cancer through the single-cell data. The specific data processing and standardization pipeline can be obtained from the original article (21).

### Exploring T-cell depletion trajectories in colon cancer

First, we evaluated the cytotoxicity score and cell exhaustion score of each cell in the scRNA-seq dataset by the “AUCell” package based on previously reported genetic markers (9, 14). Subsequently, the R package “monocle” was used to calculate and map the pseudo-time trajectories of T cells. The differentialGeneTest() function was used to calculate the characteristic genes in different trajectories. Finally, we identified the signature genes in the T cell depletion trajectories as T cell depletion markers in COAD.

### Construction of the T-cell exhaustion scoring model

In order to find independent predictive markers for COAD, we first conducted a univariate cox regression analysis for T-cell exhaustion markers. The T-cell exhaustion score (TES) was then created using LASSO regularization through 300 random iterations. After selecting a penalty factor λ, the regularization model will remove insignificant markers and generate coefficients for each TES model gene. To prevent overfitting, we set up a 5-fold cross-validation and determine the final stable TES model based on the number of builds in 300 random iterations. The final TES model was generated GAS according to the following equation:


$$TES=\sum iCoefficient\left(mRNA_{i}\right)\times Expression\left(mRNA_{i}\right)$$

In order to evaluate prognostic effectiveness, the “survcomp” program computed the C-index of the TES (22). A prediction made by the model that is more optimum and stable has a higher C-index. The independent prognostic significance of TES was thoroughly investigated using Kaplan-Meier survival analysis, univariate and multifactorial Cox regression, and time-dependent ROC (tROC) curves. High TES and low TES groups were separated by the median value of TES. Finally, to measure the chance of survival more accurately for specific patients, we created a nomogram based on TES and other clinical characteristics.

### Cell culture

The normal human colonic epithelial cell line NCM460 and the human colon cancer cell lines SW460 and SW48 were bought from Bioss, China. All cells were grown in DMEM media with 10% FBS in a 37°C cell incubator with 5% CO2.

### qRT-PCR

We then used qRT-PCR to assay patient tissues and COAD cell lines to assess TXK level. ChamQ Universal SYBR qPCR Master Mix was used to run each real-time PCR experiment (Vazyme, China). Using GAPDH as a control, the amplified PCR products were measured and standardized.

### Cell proliferation detection

For the transfection of siRNA in this work, LipofectamineTM 2000 Transfection Reagent (Invitrogen, USA) was used. Cell Counting Kit-8 kit was used to measure the proliferation rate of COAD cells (Bioss, China). At a density of around 1500 cells per well, the digested single-cell solution was injected in 96-well plates. Three wells from each group were chosen at random at 0, 12, 24, 48, and 72 hours. Then, 10 mL of the Cell Counting Kit-8 reagent was added, and the wells were incubated at 37°C for two hours. identification of 450 nm absorbance values.

### Transwell cell invasion analysis

We used a Transwell kit (Merck Millipore, USA) with a pore size of 8um to detect the degree of invasion of different SW460 cells. Briefly, SW460 cells were inoculated in the upper chamber of a 24-well plate, and DMEM medium containing 20% FBS was added dropwise in the lower chamber. Cells in the upper layer of transwell chambers were wiped off with a cotton swab after incubation for 32 h at 37°C in an incubator. The invading cells were stained with 0.1% crystal violet staining solution (Solarbio, China) and counted using ImageJ software after microscopic visualization.

### Assessment of immune heterogeneity between TES subgroups

In each COAD sample, we calculated the relative abundance of 22 different immune cell types using the “CIBERSORT” program (23). The “ESTIMATE” system evaluated the samples’ immunological score and tumor purity (24). The ssGSEA algorithm of the “GSVA” software was then used to evaluate the activity of the relevant immunological pathways. The changes in the expression of 6 classical immunological checkpoints between subgroups were then examined.

### Dissecting genomic alterations between subgroups

The “maftools” package was used to handle the maf files, and it computed the amount of nonsynonymous mutations for each patient (25). The variations in top 20 mutated genes across subgroups were next examined using OncodriveCLUST algorithm. We also used the “Sigminer” package to extract significant mutation signatures from the maf files for different subgroups and compared the mutation signatures with the COSMIC database (26). Finally, Gistic2.0 was used to process CNV data and count amplicons and deletions according to a threshold of 0.2. The “ggplot2” package was used for visualization.

### Assessment of chemotherapy applications for TES

Three medications routinely used in COAD (5-FU, Cisplatin, and Camptothecin) were initially predicted using “pRRophetic,” which was built on the GDSC database (27). IC50 values were estimated by ridge regression, with lower IC50 values indicating higher sensitivity. Since the differentially expressed genes between the high and low TES subgroups were thought to represent potential therapeutic targets, we uploaded the Top150 up- and down-regulated genes to the CMap database (https://clue.io/) to investigate prospective small molecule compounds. Additionally, to revealing the biomolecular pathways that medications target, it may infer pharmaceuticals based on gene expression patterns.

### Predicting immunotherapy response

We calculated the Immunophenoscore (IPS) of patients based on the genetic profile of different immune cell phenotypes (28). A higher IPS indicates an active immune response and a higher response to immunotherapy. We used the TIDE method to mimic the tumor immune escape mechanism in order to forecast how each patient would respond therapeutically to immune checkpoint inhibitors (29). In addition, we collected two well-established immunotherapy cohort, Imvigor210, which contained 298 patients with complete follow-up information who received anti-PD-L1 immunotherapy for uroepithelial cancer (30). And Liu David. cohort, which contained 121 patients who received anti-PD-1 immunotherapy for melanoma (31). In order to evaluate the TES’s immunotherapy prediction capability, the transcriptome data from the Imvigor210 cohort and Liu David. cohort were utilized to build the TES based on the same methodology.

### Bioinformatics and statistical analysis

Fisher’s exact test was used to find proportional differences, the Wilcoxon test or T-test to determine group differences, the Kaplan-Meier plotter to produce survival curves, and the log-rank test to detect differences in survival. The R package “survivalROC” was used to plot time-dependent ROC curves (tROC). Using the R package “survival,” univariate and multivariate Cox regressions were carried out. The nomogram and calibration curves were plotted using the R package “rms”. The prediction power of several variables on the outcome of immunotherapy was evaluated using the R package “pROC”. If not mentioned differently, two-tailed p-values 0.05 were regarded as significant. Every analysis was done using the R software (Version 4.1.0).

## Results

### Exploring T-cell exhaustion trajectories in colon cancer

**Figure 1:**
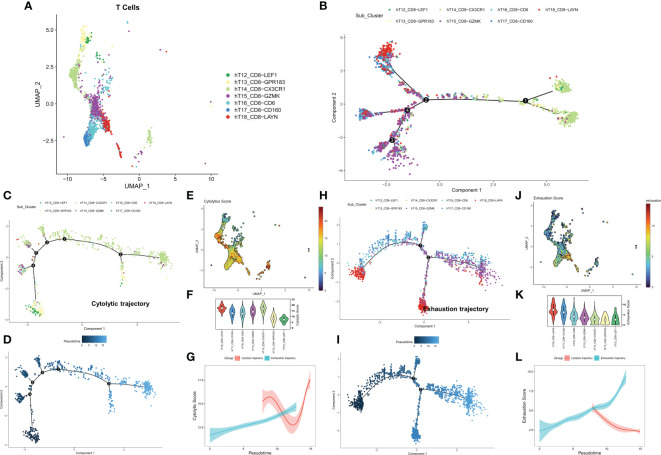
Exploring the trajectory of T-cell exhaustion in COAD. **(A)** The landscape of 7 CD8+ T cell subtypes in GSE146771. **(B)** Overall pseudo-time differentiation trajectory of seven T cell subtypes. **(C, D)** The pseudo-time differentiation trajectory of cytolytic T cell subtypes. **(E)** Density of cytolytic scores of different T cell subtypes. **(F)** Comparison of cytolytic scores of different T cell subtypes. **(G)** Cytolytic scores for different differentiation trajectories. **(H-J)** The pseudo-time differentiation trajectory of exhaustion T cell subtypes. **(J)** Density of exhaustion scores of different T cell subtypes. **(K)** Comparison of exhaustion scores of different T cell subtypes. **(L)** Exhaustion scores for different differentiation trajectories.

We first identified seven CD8+ T cell subtypes based on the cell annotation in the original article (**Figure 1A**). The overall differentiation trajectory of CD8+ T cells was then identified by monocle algorithm (**Figure 1B**). The results showed that CD8-CX3CR1 was at the beginning of the trajectory, while CD8+ T cells with high expression of the exhaustion marker LAYN were distributed at the end of the trajectory. Subsequently, we differentiated two different developmental trajectories based on the cell exhaustion fraction and cytolytic fraction. Among them, the cytolytic trajectory was mainly composed of CD8-CX3CR1 cells with CD8-LEF1 and CD8-GPR183 at the beginning (**Figures 1C, D**). We found the strongest cytolytic activity of the CD8-CX3CR1 (**Figures 1E, F**). Over time, the exhaustion score showed an increasing trend while the cytolytic score showed a decreasing and then increasing trend (**Figure 1G**). The cell exhaustion trajectory was mainly composed of CD8-LAYN and CD8-GZMK, with CD8-LAYN distributed at the beginning and the end of the trajectory and CD8-GZMK mainly distributed at the end of the trajectory (**Figures 1H, I**). Subsequently, we found the highest exhaustion score of CD8-LAYN, which confirmed the reliability of the exhaustion trajectory (**Figures 1J, K**). Over time, the cell exhaustion score increased while the cytolytic activity decreased (**Figure 1L**). Finally, we identified 477 ordered genes in the cell exhaustion trajectory as the T-cell exhaustion markers in COAD.

### Dissecting key T-cell exhaustion genes in COAD

**Figure 2:**
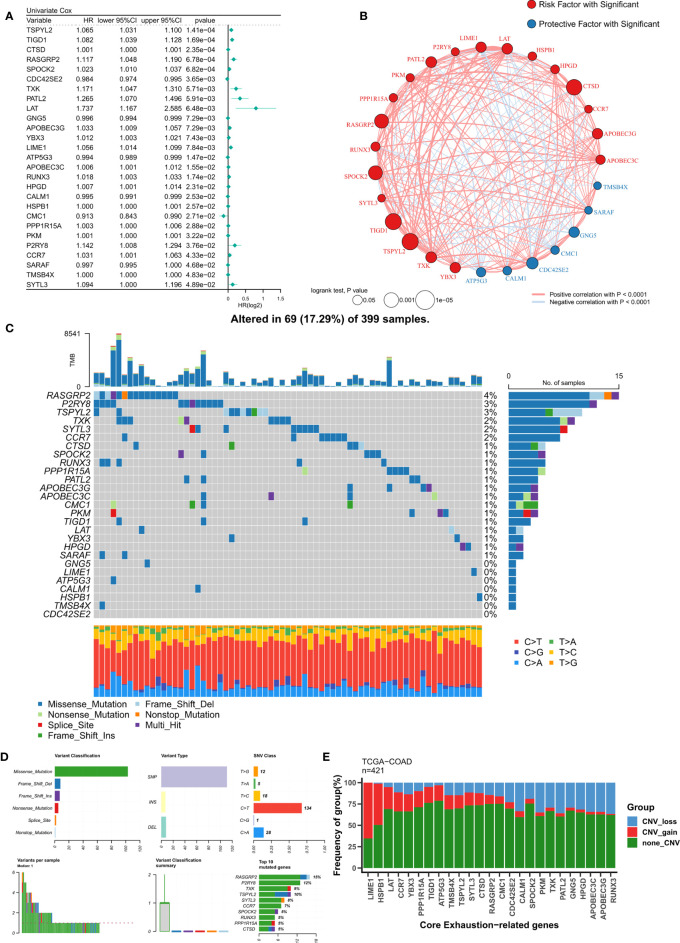
Identification of ordered T-cell exhaustion indicators in TCGA-COAD. **(A)** Univariate Cox regression identified 27 key T-cell exhaustion indicators with prognostic efficacy. **(B)** The correlation network of 27 key T-cell exhaustion indicators. **(C)** The landscape of somatic mutation of 27 key T-cell exhaustion indicators. **(D)** The summary of somatic mutation of 27 key T-cell exhaustion indicators. **(E)** The summary of CNV status of 27 key T-cell exhaustion indicators.

Based on a P<0.05 criterion, 27 T-cell exhaustion genes were discovered (**Figure 2A**). A correlation network for these 27 genes was constructed and the results indicated that most of them were positively correlated (20/27) (**Figure 2B**). In the TCGA-COAD cohort, the mutation landscape of these 27 genes was shown in **Figure 2C**. The gene with the greatest frequency of mutations is RASGRP2, and missense mutations are the most common form of mutation (**Figure 2D**). Finally, we summarized the CNV events of the 27 key genes (**Figure 2E**). The results showed that prevalent CNV events occurred in most genes, the highest amplification frequency was LIME1, and the highest deletion frequency was RUNX3.

### Construction of T-cell exhaustion score

**Figure 3:**
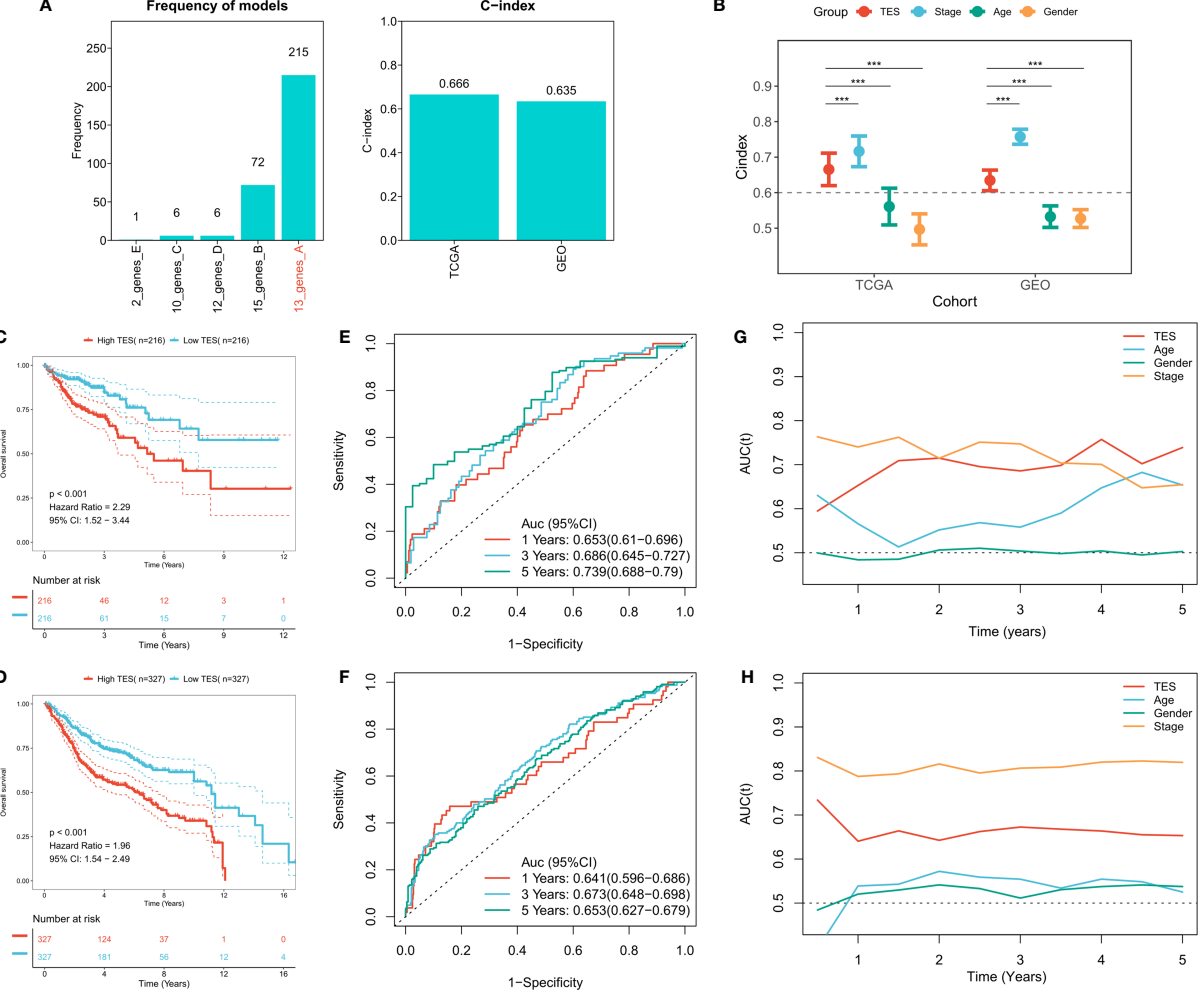
Construction of the T-cell exhaustion scoring model. **(A)** Iterative LASSO regression to select the most stable prognostic model. left: frequency of different gene pairs in LASSO models; right: C-index of the best combination in TCGA and GEO datasets. **(B)** Comparison of C-index differences between TES model and clinical characteristics. ***: P<0.001. **(C)** Kaplan-Meier survival curve of patients with high and low TES in TCGA dataset. **(D)** Kaplan-Meier survival curve of patients with high and low TES in the meta-GEO dataset. **(E)** ROC curves of TES at 1, 3, and 5 years in the TCGA dataset. **(F)** ROC curves of TES in the meta-GEO dataset at 1, 3, and 5 years. **(G)** tROC curves of TES in 5 years in the TCGA dataset. **(H)** tROC curves of TES in 5 years in the GEO dataset.

To construct a more robust TES model, we enrolled 27 T-cell genes with independent prognostic efficacy and performed 300 iterations of LASSO regression to retrieve the most robust model. The results showed that the model containing 13 genes was the most robust TES model (215/300) (**Figure 3A**). Good predictive efficacy was demonstrated in both TCGA and meta-GEO cohorts (C index: 0.666 for TCGA; 0.635 for GEO) (**Figure 3A**). Compared to the commonly used clinical indicators, TES was slightly weaker than stage but better than age and gender (**Figure 3B**). In many COAD cohorts, survival analysis revealed that patients in the high TES group had worse results than its rival (**Figures 3C, D**). Roc analysis showed that TES had acceptable performance in the TCGA cohort (ROC>0.65, **Figure 3E**), while a favorable effect was also observed in the GEO cohort (ROC>0.65, **Figure 3F**). The tROC results showed that TES had an effective performance in predicting survival within five years in both cohort (**Figures 3G, H**).

### Analysis of the predictive efficacy and independence of TES

**Figure 4:**
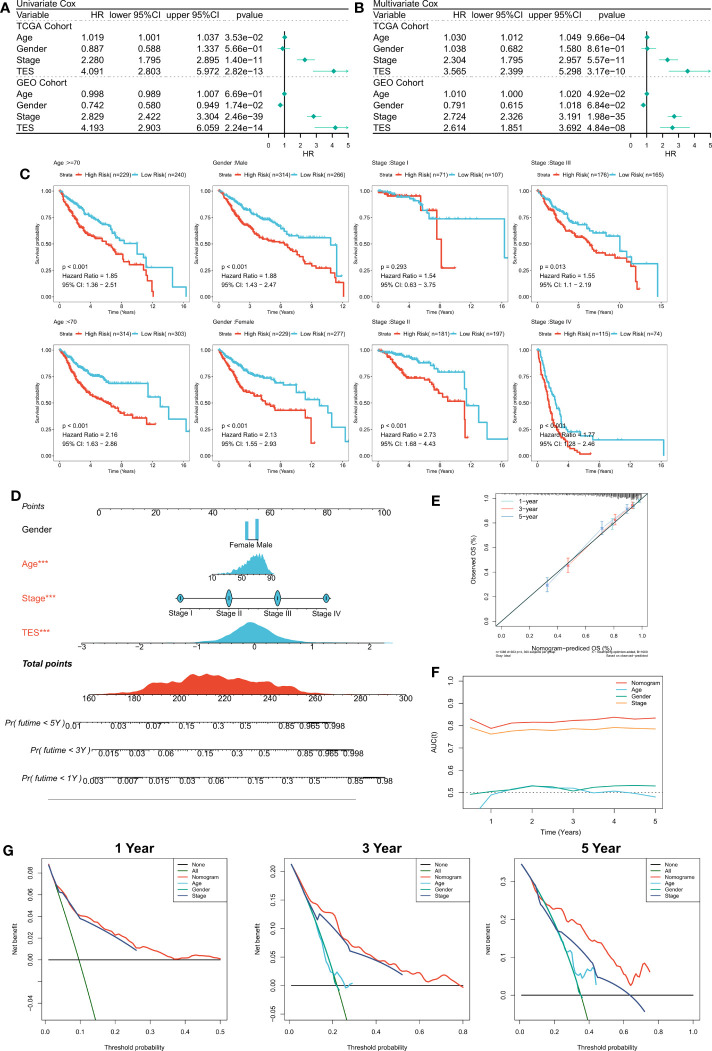
Verifying the independence and robustness of TES. **(A)** Univariate COX regression analysis of OS in TCGA and GEO datasets. **(B)** Multivariate COX regression analysis of OS in TCGA and GEO datasets. **(C)** The subgroup analysis of TES in the whole cohort. **(D)** Nomogram based on TES and clinical characteristics. **(E)** Calibration curve of Nomogram. **(F)** tROC curve of Nomogram and clinical characteristics. **(G)** The DCA curves of Nomogram and clinical characteristics at 1, 3, and 5 years.

First, we used univariate and multifactorial Cox regression to investigate the relationship between TES and patients’ clinical variables (e.g., age, gender, and stage). In the training and validation cohorts, univariate Cox regression indicated that TES was an independent predictor (p 0.05) (**Figure 4A**). In both the training and validation cohorts, multifactorial Cox regression showed that TES remained an unfavorable predictor of OS (P<0.05) (**Figure 4B**). In addition, subgroup analysis showed that TES performed best in predicting prognosis in all subgroups of patients, especially those with advanced tumors in all age groups (**Figure 4C**). Therefore, TES could be a trustworthy prognostic indicator of OS in COAD patients. We then created a Nomogram to more accurately measure the risk assessment of COAD patients (**Figure 4D**). The calibration curve of Nomogram showed high stability and accuracy after 1, 3 and 5 years (**Figure 4E**). tROC study showed that Nomogram model performed better than TES alone (**Figure 4F**). Finally, DCA analysis showed that the nomogram model had the best decision effectiveness after 1, 3 and 5 years (**Figure 4G**).

### Cellular experimental validation of key TES model indicator

**Figure 5:**
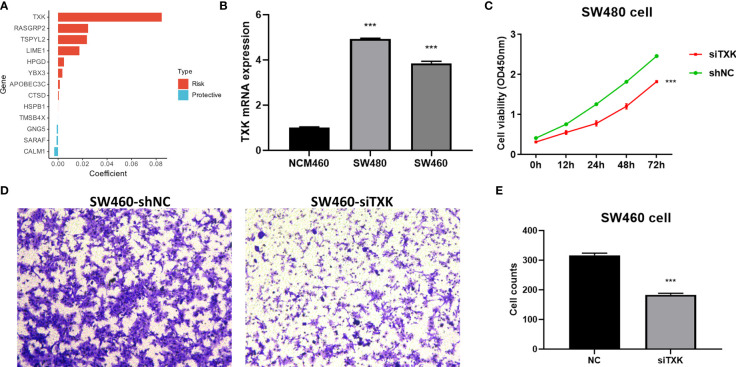
Cellular experiments to verify the malignancy of TXK. **(A)** Gene coefficients in the TES model showed that TXK was the most potent risk factor. **(B)** Differential mRNA expression levels of TXK in NCM460, SW480, and SW460 cell lines by qPCR. **(C)** Cell proliferation of SW480 cells transfected with TXK siRNA or siNC. **(D)** Transwell assay of invasive ability of SW460 cells transfected with TXK siRNA or siNC. **(E)** Cell counting of SW460 transfected with TXK siRNA or siNC in transwell cells. *** P<0.001.

We extracted risk coefficients for each indicator in the final TES model, and the results showed that TXK was the most potent risk factor (**Figure 5A**). We sought to explore whether TXK affects the malignant activity of tumor cells to influence prognosis through cellular experiments. We first found that the mRNA expression level of TXK was increased in COAD cell lines compared to normal colonic epithelial cell lines (**Figure 5B**). We then found the reduced proliferative activity of cells after knockdown of TXK in SW480 cell line by CCK8 kit (**Figure 5C**). After knockdown of TXK in SW460, invasive cells in transwell cells were reduced (**Figure 5D**). By counting the invading cells, we found that the degree of invasion of SW460 cells was significantly reduced after knockdown of TXK (**Figure 5E**).

### Low TES is associated with abundant immune infiltration

**Figure 6:**
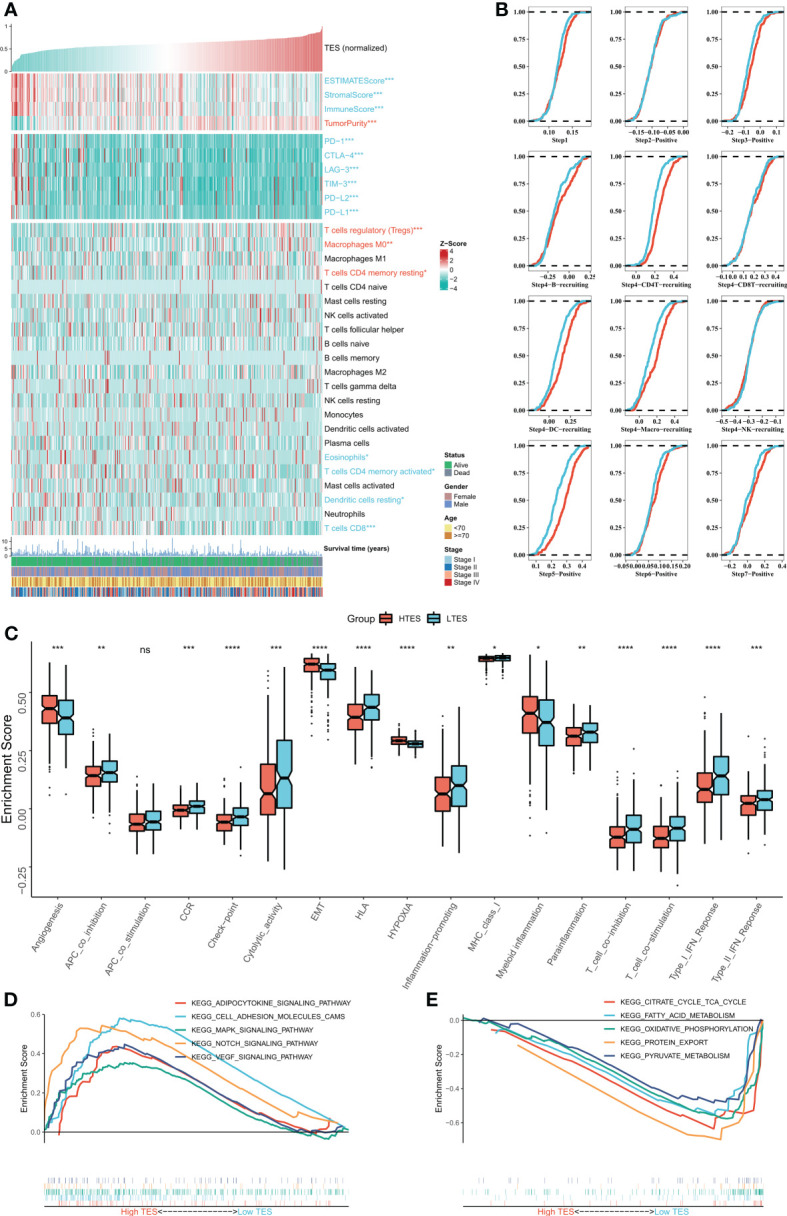
Dissecting the immune microenvironment of different TES groups. **(A)** The heat map shows the distribution of Estimate score, immune checkpoint expression and immune cell abundance among different TES groups in TCGA-COAD cohort. **(B)** Differences in tumor immune cycle among different TES groups in the TCGA-COAD cohort. **(C)** Box plots showing the immune-related pathway activity between different TES groups. *: P<0.05; **: P<0.01; ***: P<0.001; ****: P<0.0001. **(D)** GSEA analysis revealed 5 enriched pathways in the high TES group. **(E)** GSEA analysis revealed 5 enriched pathways in the low TES group. ns, not significant.

We then dissected the tumor immune microenvironment of TES. Estimate results revealed more tumor purity in the high TES group, while the low TES group had better estimate and immunological scores (**Figure 6A**). Further we found elevated expression of six typical immune checkpoints (PD-1, CTLA-4, LAG-3, TIM-3, PD-L2, and PD-L1) in low TES (**Figure 6A**). We also found increased enrichment of CD8+ and CD4+ T cells in the low TES group and increased infiltration of Tregs and M0 macrophages in the high TES group (**Figure 6A**). Subsequently, we examined the differences in immune recycling cycles between the two subgroups, and the results showed an increased recruiting of CD4+ T, DC, and macrophages in the low TES group, accompanied by an increased promotion of antitumor immunity in step V (**Figure 6B**). We then assessed immune-related pathway activity using ssGSEA. The findings indicated that the low TES group had a considerable enrichment of most immune-related pathways (**Figure 6C**). Finally, GSEA results showed significant enrichment of cell adhesion, MAPK, NOTCH and VEGF signaling pathways in the high TES group (**Figure 6D**). In contrast, the TCA cycle, fatty acid metabolism, protein export, and oxidative phosphorylation pathways were significantly enriched in the low TES group (**Figure 6E**). Therefore, our hypothesis was that CD8+ and CD4+ T cells enhanced anti-tumor immunity in the low TES group, but Tregs reduced anti-tumor immune responses in the high TES group.

### Correlation of TES with genomic alterations

**Figure 7:**
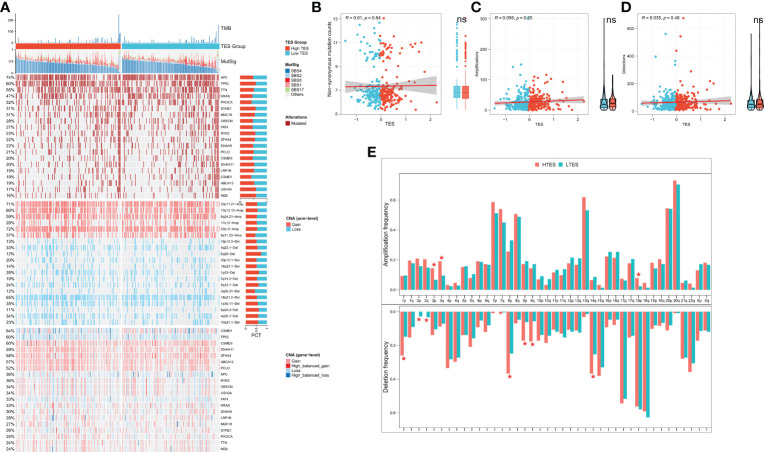
TES distinguishing genomic alteration patterns in COAD patients. **(A)** Genomic alterations landscape between different TES groups in the TCGA-COAD cohort, from top to bottom: TMB, mutational signatures, single-nucleotide mutations of top 20 driver mutated genes, CNVs of chromosomal segments, and CNVs in top 20 driver mutated genes. **(B)** Correlation of TES with Non-synonymous mutation counts. **(C)** Correlation between TES and total amplification number. **(D)** Correlation between TES and total deletion number. **(E)** CNV differences between different TES subgroups on the chromosome arms. *: P<0.05. ns, not significant.

We then analyzed genome-wide data of the TCGA-COAD to decipher the genomic alteration status of different TES groups. The overall mutation profiles among the TES subgroups (including TMB, mutation signatures, SNP, and CNV) are shown in **Figure 7A**. Between the two groups, we didn’t detect any discernible differences in TMB (**Figure 7B**). In addition, we discovered no discernible variations in high frequency mutations across groupings except for TP53 (**Figure 7A**). We then detected no change in the total chromosomal amplification and deletion number between the two categories (**Figures 7C, D**). However, we discovered that the high TES group had greater gain and loss events in both arm and gene level (**Figure 7A**), while the low TES group had more deletions on 2p and 2q arms (**Figure 7E**).

### Patients with low TES are more sensitive to chemotherapy

**Figure 8:**
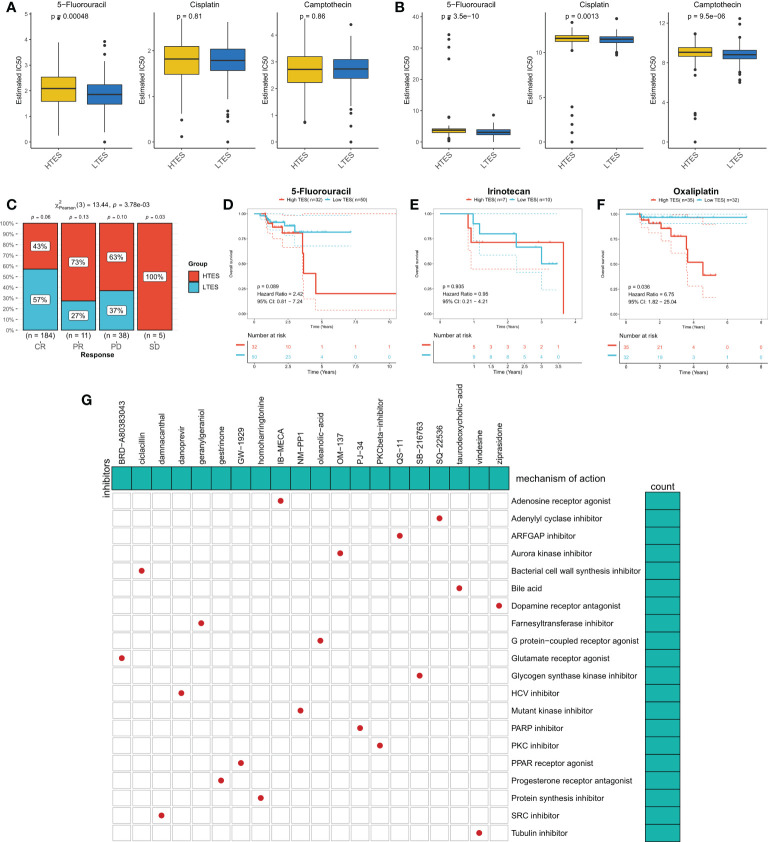
TES can predict chemotherapy. The IC50 values of the three commonly used drugs (5-Fluorouracil, Cisplatin, and Camptothecin) in the **(A)** TCGA cohort and **(B)** meta-GEO cohort were predicted based on the GDSC database. **(C)** Remission rates of different TES patients after receiving chemotherapy. **(D)** Kaplan-Meier survival curves for patients treated with 5-Fluorouracil in different TES groups. **(E)** Kaplan-Meier survival curves for patients treated with Irinotecan in different TES groups. **(F)** Kaplan-Meier survival curves for patients treated with Oxaliplatin in different TES groups. **(G)** Prediction of TES-related small molecule compounds as well as target pathway from the CMap database.

We proposed the hypothesis that TES might predict the response to chemotherapy in COAD patients given the disparities in biological function and CNV across different TES patients. On the basis of the GDSC database, we first assessed the IC50 of frequently used chemotherapeutic agents for COAD in various TES groups. The findings revealed that patients with low TES were more responsive to 5-Fluorouracil (**Figures 8A, B**). Contrarily, patients in the validation group with low TES were more responsive to Cisplatin and Camptothecin (**Figure 8B**). We examined the response of patients with different TES to chemotherapy in the TCGA dataset. The results showed that patients in the low TES group had a greater chance of complete and partial remission. In contrast, the proportion of patients with disease progression was higher in the high TES group (**Figure 8C**). Survival analysis showed better survival in the low-TES group of COAD patients receiving 5-FU and oxaliplatin, especially in those receiving Oxaliplatin (**Figures 8D, F**). In contrast, the survival difference between the different groups of COAD patients receiving Irinotecan was not significant, which may be due to the small sample size (**Figure 8E**). In conclusion, we speculate that patients with low TES are more suitable for treatment with 5-FU and platinum-based chemotherapeutic agents. Finally, we retrieved 20 small molecule compounds that may target TES through the Cmap database (**Figure 8G**)

### Inferring immunotherapy response

**Figure 9:**
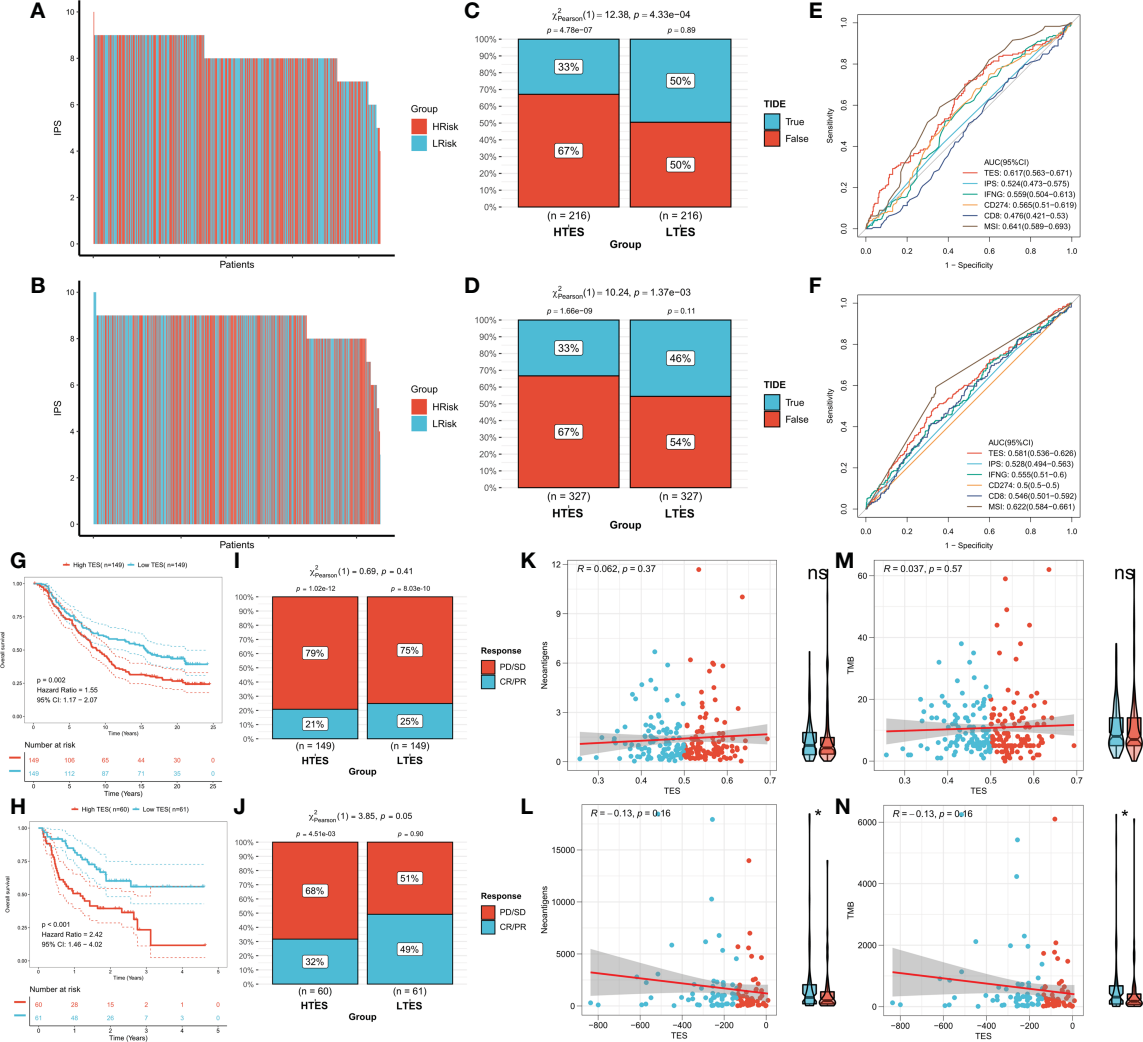
TES can predict immunotherapy. The IPS of individual COAD patients in the **(A)** TCGA cohort and **(B)** meta-GEO cohort. TIDE algorithm predicts response rates to immune checkpoint inhibitors for patients in the **(C)** TCGA cohort and **(D)** meta-GEO cohort. ROC curve shows the predictive efficiency for the response rate to immunotherapy by TES and other indicators in the **(E)** TCGA cohort and **(F)** meta-GEO cohort. **(G)** KM survival curves for patients in the high- and low-TES groups in IMvigor210 cohort. **(H)** KM survival curves for patients in the high- and low-TES groups in Liu David cohort. Remission rates of different TES patients after immunotherapy in the **(I)** IMvigor210 cohort and **(J)** Liu David cohort. Scatter plot and box plot show the correlation of TES with **(K)** neoantigens; **(M)** TMB in IMvigor210 cohort. Scatter plot and box plot show the correlation of TES with **(L)** neoantigens; **(N)** TMB in Liu David cohort. *: P<0.05. ns, not significant.

We hypothesized that the low TES group would respond more strongly to immunotherapy because they have a more powerful antitumor immune response. First, we determined the unique IPS of each patient and found that individuals in the low TES group had greater IPS in both cohorts (**Figures 9A, B**). The TIDE algorithm was then used to predict the response of patients in the TCGA and GEO cohorts to immune checkpoint inhibitors, and the results showed that patients with low TES in both cohorts had a higher response rate to immunotherapy (**Figures 9C, D**). The efficacy of TES in the TCGA and GEO cohorts was only lower than MSI with reliable predictive efficacy compared to other indicators of immune efficacy (**Figures 9E, F**). Subsequently, we worked in two real-world immunotherapy cohorts (Imvigor210 and Liu David). The results showed significantly better survival in the low-TES group in both cohorts (**Figures 9G, H**). Moreover, patients in the low-TES group had higher remission rates in both cohorts (**Figures 9I, J**). Subsequently, we found no significant association between TES and neoantigens in both cohorts (**Figures 9K, L**). Although there was also no significant association between TES and TMB in both cohorts (**Figures 9M, N**), TMB was significantly higher in the low-TES group in the Liu David cohort (**Figure 9N**).

## Discussion

Advanced COAD is extremely malignant and often has a poor prognosis due to treatment resistance (32). The immune system is involved in resisting the proliferation and invasion of malignant cells in COAD process, and T-cell depletion is one of the main causes of diminished antitumor immunity (33). In addition, targeted T-cell depletion is emerging in the field of cancer immunotherapy (11). To provide a new approach to the treatment and prognosis of advanced COAD, we aimed in this study to analyze the trajectory of T-cell depletion in COAD patients.

In this work, by analyzing scRNA-Seq data of TCGA-COAD, we differentiated cytolytic trajectories and exhaustion trajectories of CD8 T cells to comprehensively identify the major T-cell exhaustion indicators. We identified a total of 477 ordered genes for exhaustion trajectories and subsequently identified 27 effective T-cell exhaustion markers by one-way Cox regression. We observed a significant positive correlation between them, suggesting a potential mutual regulation between them. The primary transcriptome regulator for all core genes was CNV. we built a 13-gene T-cell depletion score (TES) model based on these 27 core genes using iterative LASSO regression. We first report the prognostic efficacy of a systematic TES model for COAD patients compared to previously presented T-cell exhaustion markers (HAVCR2, ENTPD1, LAYN, and LAG3). We confirmed that TES is a strong prognostic indicator of OS in COAD patients and that TES works well in different COAD cohorts.

We next attempted to comprehend the molecular rationale behind TES, which is the key path of the dynamic processes of T cell differentiation in malignancies and is involved in the proliferation and spread of tumor cells. By comparing the two groups from multiple perspectives, including immune cell infiltration, immunological pathways, and immune checkpoints, we were able to explore in more detail the differences in the immune environment between the different TES groups. the results of ESTIMATE showed that the immune rating and Estimate composite score were higher in the low TES group while the tumor purity was higher in the high TES group. In addition, in the low TES group, we later found enhanced expression of six typical immune checkpoints, suggesting that patients with low TES may benefit more from treatment with immune checkpoint inhibitors (34). The tumor immune cycle system is characterized by most of the processes of antitumor immunity (35), and we found increased activity of the recruiting process of active immune cells (including T cells, DCs, and macrophages) as well as the positive regulatory processes of antitumor immunity in the low-TES group. Most immune-related pathways were also considerably elevated in the low TES group, supporting lower T-cell exhaustion in the low TES group, and pointing to a more potent and aggressive antitumor immune response in this group (35). Notably, there was a statistically significant increase in the number of Treg cells infiltrating the high TES group. This finding may have stifled the immune environment and antitumor immune response in patients with high TES, leading to a poorer prognosis (36).

Considering the significant relevance of genomic mutations for the course of tumor progression and treatment response, especially immunotherapy response, we then analyzed genome-wide data to explore the differentiation of genomic variation patterns of TES for individual patients. We were not able to detect significant TMB differences between TES subgroups. However, we found that the TP53 gene was significantly more frequently mutated in the high TES group. Previous studies have demonstrated that TP53 is the most frequently mutated tumor suppressor gene during tumor progression (37). Loss of function or dominant inactivation of wild type p53 is also frequently detected in patients with colon cancer (38, 39), which is consistent with our results. Our study indicates that increased mutations in TP53 may lead to a higher malignancy of tumor cells in the high TES group, resulting in a worse prognosis. Finally, we discovered more CNV occurrences in the group with high TES. Additionally, it has been shown that CNV has a significant role in the regulation of genes that affect drug response and metabolism, which in turn speeds up the development of anticancer drug resistance and results in treatment failure and disease recurrence (40, 41). As a result, we deduce that patients with low TES are suited for chemotherapy, whereas those with high TES are resistant to it. We confirmed the resistance to chemotherapy in patients with high TES through drug sensitivity data provided by the GDSC database. We found that low TES patients were more susceptible to 5-FU. In addition, survival analysis in the TCGA cohort also demonstrated an elevated remission rate for chemotherapy in patients with low TES, especially for oxaliplatin and 5-FU treatment. For high-risk COAD patients based on TES, we also screened for potential therapeutic targets and found comparable small-molecule drugs. Finally, we identified the 20 most probable small molecule compounds.

Finnaly, we made a prediction that, from a variety of angles, people with low TES are more susceptible to immunotherapy. Moreover, IPS was greater among COAD patients with low TES, indicating that these patients would respond to immunotherapy better (28). The TIDE algorithm also demonstrated that individuals with low TES had greater rates of immune checkpoint inhibitor response (e.g., anti-PD-1, anti-PD-L1, and anti-CTLA-4) (29). Additionally, TES was more reliable than traditional predictors in predicting immunotherapy response. It is worth noting that the predictive efficacy of TES is not higher than that of MSI, which has been shown in numerous studies to be a reliable predictor of immunotherapy in colon cancer and is now being tested in clinical practice to assist in the prognosis of patients with COAD. Therefore, although TES does not show a leading advantage, it can be used as a complement to MSI in clinical practice (42–44).

In addition, two real-world cohorts used for validation confirmed our predicted results for immunotherapy sensitivity. In both the Imvigor210 and Liu David cohorts we found that the low TES group exhibited better survival rates. However, we did not find a significant association between TES and the number of detected neoantigens and TMB in these two cohorts. Previous studies have shown that TMB and neoantigens are indicators of a strong relationship with immunotherapy efficacy and can be used to predict benefits for patients. However, TES in our results exhibited independent predictive accuracy for immunotherapy. More insight into the specific regulatory mechanisms is needed in future studies (45, 46).

This study still contains some limitations. The study only contains two non-COAD immunotherapy RNA-seq data, which is due to the scarcity of data in this field. We hope to collect more immunotherapy sequences or platform data for COAD in the future to further validate the predictive accuracy of TES for immunotherapy. In addition, genomic regulation is a large field, and we have only focused on a portion of mRNAs and may have neglected data from some other regulatory genomes. Finally, the mechanism of how TES affects biological function as well as the phenotype is unclear. However, we synthesized the results of functional enrichment analysis to make reasonable speculations, which is an inspiration for future mechanistic studies.

## Conclusions

In this study, we identified possible depleted CD8+ T cell differentiation trajectories in COAD patients and developed a TES model to quantify the level of T cell depletion in the tumor microenvironment. Patients with lower TES responded more strongly to chemotherapy and immunotherapy and had a better prognosis. This finding not only advances the development of cancer genetics and immunotherapy but also provides new perspectives on the clinical treatment of colon cancer and innovative immunotherapy strategies.

## Data availability statement

The datasets presented in this study can be found in online repositories. The names of the repository/repositories and accession number(s) can be found within the article/supplementary materials.

## Author contributions

XS performed the data analyses and wrote the manuscript. XZ contributed significantly to analysis and manuscript preparation. LL helped perform the analysis with constructive discussions. BL and LW contributed to the conception of the study. All authors contributed to the article and approved the submitted version.
